# Seeding the idea of encapsulating a representative synthetic metagenome in a single yeast cell

**DOI:** 10.1038/s41467-021-21877-y

**Published:** 2021-03-11

**Authors:** Ignacio Belda, Thomas C. Williams, Miguel de Celis, Ian T. Paulsen, Isak S. Pretorius

**Affiliations:** 1 Department of Genetics, Physiology and Microbiology, Complutense University of Madrid, Madrid, Spain; 2 Department of Molecular Sciences, and ARC Centre of Excellence in Synthetic Biology, Macquarie University, Sydney, Australia

**Keywords:** Synthetic biology, Microbial ecology

## Abstract

Synthetic metagenomics could potentially unravel the complexities of microbial ecosystems by revealing the simplicity of microbial communities captured in a single cell. Conceptionally, a yeast cell carrying a representative synthetic metagenome could uncover the complexity of multi-species interactions, illustrated here with wine ferments.

## Searching for consilience in complex adaptive ecosystems

The natural complexity of adaptive ecosystems—patterned by diversity, interconnections and dynamic processes—served as a rich source of ecological thought and scholarship throughout the history of science. A Newtonian-Cartesian approach to ‘complicated’ problems is an effective way to unravel phenomena and predict the outcome of interactions if one can analyse, measure and account for all the parts of a system. However, in ‘complex’ ecosystems even simple interactions can have comprehensible, but unexpected and unpredictable outcomes. Only integrative systems science—uniting ideas, evidence, principles and knowledge emerging from different strands and facets of research—can ground mathematical models, findings and conclusions about the interactions among organisms and their biophysical environment (including both biotic and abiotic components) in the reality of complex ecosystems.

The successful application of consilience in science compels us to keep advancing the frontiers of knowledge and, in unison with other disciplinary branches, to keep searching for ‘the next big thing’ early so that we can make the application of ‘the last big thing’ usable, practicable and beneficial^1–3^. With this spirit, we propose here that contemporary synthetic biology technologies could provide an additional research prism through which researchers could gain insights into the web of interconnected networks within microbial communities that communicate, cross-feed, recombine, coevolve and coexist in, for example, specific wine-related *terroirs*^4,5^. More specifically, we would like to seed the idea of using a synthetic metagenome to capture the essence of a wine yeast community (typically composed by a few tens of yeast species) in a single cell, using *Saccharomyces cerevisiae* (the dominant fermentative yeast species of all wine fermentations) as a chassis.

## Synthesising a link between lab and life

Synthetic Biology has revolutionised research in life sciences. By combining DNA reading (sequencing), writing (synthesis) and editing technologies with the integrative functionalities of artificial intelligence and biodigital engineering^2,3^, it seems reasonable to envisage a future ‘bio-informational’ scenario where researchers, could design ecosystem-wide dynamic models and network-based analytical approaches to disentangle microbial interactions, and to assess, measure and control complex biological systems. The early signs of such a scenario are already visible and real in that, close on the heels of the creation of the first bacterial cell controlled by a chemically synthesised genome^6^, the synthesis of the first eukaryotic genome is nearing completion in the context of the Synthetic Yeast Genome (Sc 2.0 or Yeast 2.0) consortium^7,8^.

However, the complexity of life in nature goes beyond the genomics of a single species. Organisms live in communities, each occupying a niche that is essential for the functioning of an ecosystem. Therefore, systems biology—aided by synthetic genomic tools—aims to move from the study of isolated genomes or organisms, to the understanding of all the components of a certain ecological environment as an intricate network. Community- and population-level metabolic network modelling could potentially resolve ecological properties of microbiomes and possibly even identify keystone species in the maintenance of certain ecosystem phenotypes within specific environmental niches^9^. A seminal definition of this conceptual framework and an interesting methodological approach for deciphering key functionalities in microbial metabolic networks, their encoding genes and their host organisms have been proposed^10,11^.

## Untangling yeast communities with synthetic metagenomics

**Fig. 1:**
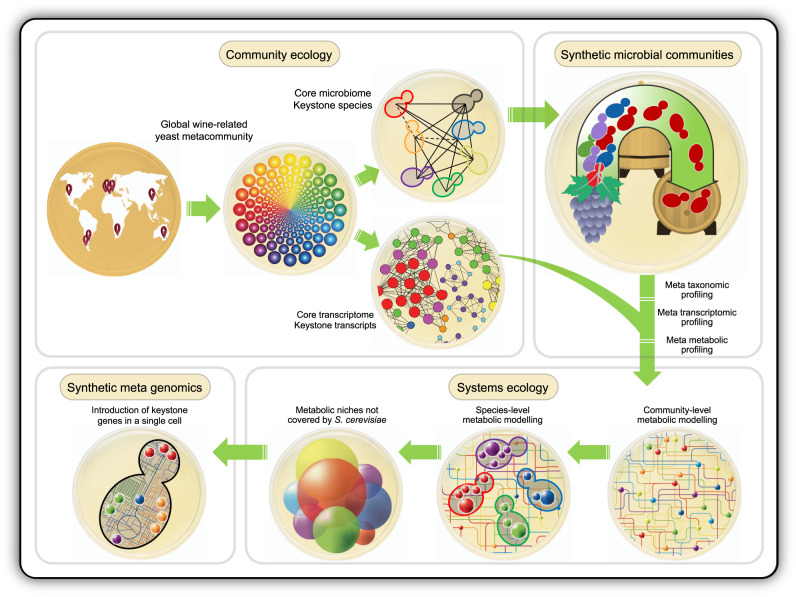
The idea of encapsulating the complexity of a natural yeast community with a representative synthetic metagenome. This concept explores the potential to reproduce a whole ecosystem’s metabolic network in a single yeast cell.

Assuming that, in the near future, the Yeast 2.0 project will successfully deliver a physiologically fit yeast cell powered by 16 man-made chromosomes, we believe that the next big thing would be to move from a synthetic *S. cerevisiae* genome to the de novo synthesis of metagenomes that represent microbial communities. Instead of designing multi-species ecological communities with desirable properties^12^, or engineering them to control the behaviours of individual species within a consortium^13^, we propose here the idea of reproducing a whole ecosystem’s metabolic network in a single cell (Fig. 1). Would a representative synthetic metagenome, encapsulated in a single yeast cell, enable researchers to reproduce and engineer microbial communities, summarising the contributions of the different members of a microbial consortium in the genome of one of their keystone members?

The culturability of yeasts in naturally fermenting grape must provides an opportunity to accurately monitor and assay the sequential succession and dynamics in model synthetic microbial communities. These scalable and highly reproducible assays should be analysed within the context of systems ecology to decipher the community-level metabolic network and the individual contribution of the different species, defining the metabolic niches^9^ required for a full-operative wine ecosystem (Fig. 1).

The idea that we propose here is not without risks, limitations and challenges. We believe that some of these challenges offer unique learning opportunities that could expand our understanding of basic and intriguing aspects of cell biology and genetics. Apart from the physical limitations of yeast cell size when hosting large amounts of additional (exogenous) genomic material (see the work by Peris et al.^14^ to get an idea of the effect of genome size on yeast cell volume), different emergent properties—from pleiotropies to epistasis and the derived metabolic trade-offs^15–17^—may arise with the introduction of each new synthetic pathway. However, all these potential pitfalls should serve to stimulate the study of basic biological aspects regarding the existence of genomic and functional incompatibilities in yeast cells, the fitness cost and dynamics of adaptation when incorporating exogenous genes or functions (from phylogenetically close or distant species), or even the limits of hoarding of ecosystem functions that might compromise the fitness, and the ecological and evolutionary stability of the yeasts.

Among the interesting ecosystems to test this idea, one could imagine different anthropic environments (food fermentations, biofuels production bioreactors, etc) where one can define a strong core microbial diversity and a well-known and bounded metabolic functionality. In particular, food fermentations (e.g. cheese, kefir, wine) are experimentally tractable ecosystems for microbiome studies^18^. Thus, as shown in Fig. 1 and as we will argue later on, here we want to propose the yeast communities of wine fermentations as an interesting model to get this idea up and running.

## Practical considerations for synthetic metagenomes

### Metabolic modelling of complex and dynamic microbial ecosystems

Understanding the structural organization of species constituting a chosen microbial community is essential to assess its biodiversity patterns, community composition dynamics and niche partitioning^19^. It is necessary to select an appropriate mathematical model to describe the community dynamics of microbial systems, as a first step for further efforts, such as the engineering of complex microbial communities with desired traits. With this aim in mind, different mathematical—linear or non-linear—models have been applied to time-resolved sequencing data (obtained from metagenomic or metataxonomic experiments), like the Generalized Lotka-Volterra (GLV) and its different variations^20^.

**Fig. 2:**
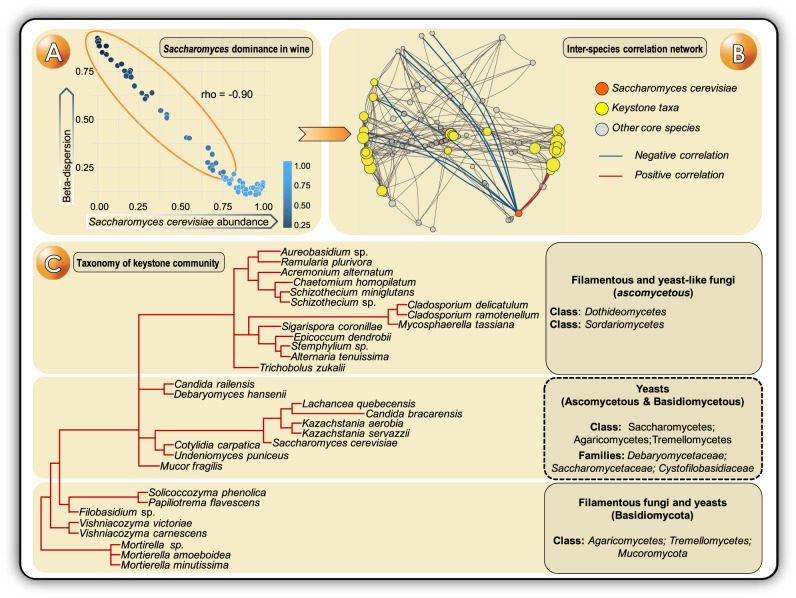
General figures about the core fungal community of wine fermentations, estimated from the regional dataset by Liu et al.^59^ (raw data publicly available at PRJNA594469). **A** As the dominant species in wine fermentations, *Saccharomyces cerevisiae* abundance has a strong impact in the diversity of fungal communities. **B** The structure of an inter-species correlation network defines some keystone taxa with a strong potential role in community functioning. Nodes in the network are sized based on their keystone score—calculated as an average of their Mean Degree, Closeness Centrality, and Betweenness Centrality values. Those nodes with higher keystone scores (>0.75) are highlighted as yellow spheres representing keystone taxa. Positive and negative correlations between the abundance of *S. cerevisiae* (orange node) and other core taxa are highlighted with red and blue edges, respectively. **C** The taxonomy of the keystone community includes both Ascomycetous and Basidiomycetous fungi including some yeasts, yeast-like and filamentous fungi. The particular insights depicted here came from a regional dataset of Australian wine fermentations, but a wider meta-analysis is needed for defining the actual global core mycobiome of wine fermentations and the expectable regional *terroir*-associated particularities.

An accurate snapshot of the physiological state of a community can be made by combining the information obtained by different meta-omics experiments. Network analysis utilises multiple layers of meta-omics data to evaluate the dynamics of complex microbial communities from a global perspective^21^. This approach can be used to model the co-occurrence patterns within a community and to unveil microbial interaction patterns and their bidirectional relationship with their habitat composition^4^. For instance, the topology of metacommunity networks reveals patterns of species interactions in a given ecosystem, giving researchers insights into how these patterns affect ecosystem structure and processes, being of great interest for closing the gap between environmental microbiomes and metabolomes^22^. The presence and distribution of modules in these networks serve as proxies for the study of microbial relationships and niche partitioning^23^, and the topological features (i.e. high mean degree, high closeness centrality and low betweenness centrality) define quantifiable thresholds for the identification of potential keystone species driving community composition and functioning^10^ (Fig. 2). Similarly, community-wide metabolic networks can be reconstructed by combining metagenomic, metatranscriptomic and metaproteomic data, thereby enabling the measurement of gene expression and protein abundances in combination with network topological features, as recently applied to identify microbial genes involved in human disease status^24^. This approach is useful to identify genes encoding key functionalities, specific functions with a pronounced effect on the functioning of the ecosystem. Similar to keystone species identification (Fig. 2), keystone genes can be predicted by the topological features of reconstructed metabolic networks, aside from having high expression levels. This framework permits the link of genes encoding key functionalities to structural and functional important community members^11^.

Biological communities, with desirable functions, can be designed by selecting species with selected traits and gradually adding more species and screening pairs forming favourable interactions. Conversely, it has been proposed that systematical exploration of combinations of species/strains could potentially maximise desired ecosystem functionalities^25^. This approach utilises microbial interaction networks (e.g. co-occurrence networks) and microbes’ ecological function as input. In this way, species are selected according to their topological position in the network, community-scale functional capability and community-scale functional balance. Selected functional core microbiomes are expected to organise whole ecosystem processes and functions^25^. These synthetic microbial communities can then be utilised to unveil the metabolic and molecular mechanisms involved in inter-species interactions. Besides, this allows the assessment of emergent metabolic behaviours and therefore, inter-species interactions modulating phenotypes of interest within microbial communities^26^.

### Synthetic genomics

Thus far, all synthetic genome projects have involved the chemical synthesis of relatively small (1–10 kb) pieces of DNA, which are then stepwise assembled into progressively larger segments using both in vitro and in vivo methods^8,27^. These methods are relatively expensive and laborious at present, however, there are two future trends that will rapidly improve the feasibility of synthetic genomics projects. The first is the increasing use of automation in molecular biology using liquid handling robotics as adopted by the Global Biofoundry Alliance (GBA), which aims to share expertise and distribute projects that focus on automation^28^. The second major trend that will continue to facilitate large synthetic genome projects is improved DNA synthesis technology and associated cost decreases. Enzymatic, template-independent DNA synthesis^29^ has the potential to make DNA synthesis sufficiently inexpensive that whole-genome synthesis may become commonplace.

Once DNA is synthesised, there are two main methods for introducing synthetic DNA and replacing the native genome. The first involves the assembly of the synthetic genome, mainly in yeast, followed by extraction and transplantation of the genome in the host cell. This method was used to construct synthetic *Mycoplasma* genomes^6^. The second method, employed by the Yeast 2.0 consortium, involves the iterative replacement of the native genome with synthetic DNA. This method carries the advantage that design changes resulting in fitness defects can mostly be identified during construction. Both methods harness the highly efficient homologous recombination machinery of yeast, alongside its capacity to harbour large amounts of heterologous DNA. In addition to yeast-mediated in vivo homologous recombination, there are several other methods that could be employed for synthetic metagenome DNA assembly. For example, there is a suite of in vitro DNA assembly methods such as Gibson, Golden Gate, and MoClo that can be used to enzymatically join synthesised or PCR amplified DNA segments larger than 20 kb for sub-chromosome assembly^30,31^. Rolling circle amplification has also been used to amplify large yeast-assembled DNA fragments for subsequent integration to the *Salmonella typhimurium* genome^32^. This approach circumvents the low yields of synthetic chromosome DNA that are typically recovered from yeast, without the size limitations of PCR. Other potential approaches to synthetic metagenome construction could involve the cloning of large environmental DNA segments or even whole chromosomes onto yeast centromeric plasmids^33^. This approach has been used to clone megabase-sized bacterial genomes in yeast^34^. Centromeric plasmids could then either be manipulated using the suite of yeast molecular biology tools, or randomly recombined in the target host cell followed by appropriate selection pressures for desired synthetic metagenome properties. Without extensive refactoring of cloned gene expression modules, this approach would limit synthetic metagenomes to expression in closely related organisms.

The largest segment of additional synthetic DNA constructed in yeast was the 1.1 Mb *Mycoplasma mycoides* genome^6^, while there are also large synthetic yeast ‘neochromosomes’ that contain functional yeast DNA. These neochromosomes represent a different class of synthetic chromosome as they are not functionally ‘inert’ like synthetic bacterial genomes in yeast, nor are they a replacement of native chromosomes. They are large extra segments of DNA that are functional in their yeast host, and are the most similar to the synthetic metagenomes that we are proposing here.

Clearly, the construction of a synthetic metagenome is well beyond the scale of any existing synthetic genome projects, but is it within the realm of possibility? In order to even start to address this question, it is first necessary to estimate the approximate size of a synthetic metagenome. One possible design specification is where (i) core genes conserved across the microbiome community involved in functions, such as transcription, translation, cell division and central metabolism, are represented by the native yeast core gene set and hence not included in the design; and (ii) the design of the synthetic metagenome encompasses the ‘auxiliary’ genes represented the broader pan-genome of the microbiome, but where there are clear orthologues conserved in more than one genome in the microbiome, only a single representative is chosen. Based on this design specification, and accounting ~1000 unique genes per species and ~1000 nucleotides per gene, here are two back of the envelope calculations. For a ‘simple’ microbial community (which can resemble the complexity of a wine yeast community) with ~50 microbial species the estimate would be ~50 Mb of synthetic DNA. For a ‘complex’ microbial community with ~1000 microbial species, the estimate would be 1000 Mb of synthetic DNA.

Another practical issue to be considered is how much additional DNA can be physically accommodated within a single *S. cerevisiae* cell/nucleus. There are natural and synthetic stable triploid and tetraploid yeast strains, with some evidence that yeasts can tolerate hexaploidy^35^, or even dodecaploidy^14^ implying a notable increase in the size of the yeast cell and nucleus. This indicates that several mega base-pairs of additional DNA can be functionally fit inside a yeast nucleus and replicated; thus assuming that a single yeast cell could at least tolerate sufficient synthetic DNA to represent a synthetic microbiome of a simple microbial community (e.g. yeasts in a wine ferment). Genetic instability may result from synthetic metagenome neochromosomes, as was observed in the recently developed six-species synthetic hybrid yeast strain^14^. Instable strains such as this can simply be cultured over time to adapt to relevant conditions. In the case of a synthetic metagenome, appropriate selection pressures would need to be developed to retain desired functions as instability elements are lost from the genome during the adaptation phase.

It is not fully clear how many additional synthetic chromosomes, or how large in size the synthetic chromosomes can be fashioned without compromising yeast fitness or genome stability. *S. cerevisiae* does seem to have some significant degree of flexibility in genome architecture, as evidenced by the fusion of its existing chromosomes into one single chromosome^36^, and by the construction of stable synthetic neochromosomes^8^.

## Functional aspects of a synthetic metagenome

To simulate the succession stages of a wine yeast ferment using a synthetic metagenome, it would be necessary to co-ordinately regulate the expression of the thousands of synthetic genes. This presents several major problems from an engineering perspective. For example, it is currently impossible to predict the required expression levels for optimal synthetic gene function, while using arbitrary native yeast promoters would dilute transcription factor pools and result in deleterious regulatory crosstalk with native processes. Furthermore, *S. cerevisiae*’s highly active homologous recombination machinery would prevent stable maintenance of synthetic chromosomes that reused identical promoter sequences.

A potential solution to these challenges is to use a library of synthetic promoter sequences that have been designed to lack repetition while retaining defined features. This was recently demonstrated through the use of an algorithm to design 1722 non-repetitive yeast promoters that span a 25,000-fold range of expression levels^37^. To ensure functionality, these unique promoter sequences retained transcription factor binding motifs, TATA boxes, and nucleosome-binding motifs. Although this ground-breaking tool would mitigate the risks of synthetic genome instability through homologous recombination, it would not solve issues associated with inappropriate expression levels or interference with endogenous regulatory networks without further promoter-library engineering. Similarly, libraries of synthetic 5′ untranslated regions (UTRs) have been engineered in *S. cerevisiae*^38,39^ with machine learning applied to understand their function and design further sequences^40^. These tools provide the most feasible mechanisms for arbitrarily assigning promoter sequences to synthetic genes in large designer chromosomes. Synthetic libraries have also been designed for terminator sequences^41–43^, which pose fewer challenges due to the structural nature of their function and relatively fewer interactions with regulatory proteins.

A significant issue is the functional expression of tens of thousands of heterologous genes in *S. cerevisiae*. Based on large-scale gene expression studies for structural genomics, the success rate for heterologous expression of individual microbial genes is ~50%^44^. In these studies, the genes are being expressed at very high levels for the purpose of protein purification, so it seems reasonable to expect higher success rates of heterologous expression with more ‘natural’ levels of gene expression.

This raises the question then of how to undertake phenotypic, fitness and expression testing for tens of thousands of genes in parallel. Phenotypic testing to check the function of the full repertoire of synthetic genes does not appear to be feasible, even with the current or projected test capabilities of existing biofoundries. In addition, a large percentage of the synthetic genes would be of unknown function, or may have functions that would not be straightforward to test in a heterologous system. On the other hand, gene expression at an RNA or protein level can be easily tested in high throughput through transcriptomic or proteomic approaches. Fitness defects resulting from any synthetic DNA regions can be easily detected using a battery of growth assays relevant to the intended environment for synthetic metagenome strain^45–47^. Thus, expression and fitness testing could be carried out for the full repertoire of synthetic genes, even if phenotypic testing would remain impractical in the foreseeable future.

If a modular, sequential build strategy was used for the novel synthetic chromosome(s) analogous to that developed for Yeast 2.0, then any deleterious fitness consequences or expression problems for particular genes could be relatively easily isolated. The design could potentially then be tweaked by replacing the non-expressed/problematic genes with orthologues from close relatives. However, there could be a significant scale problem if that involved hundreds of orthologues for thousands of genes, and testing their function. It may be simpler, if less satisfying, to eliminate problematic genes from the synthetic chromosome designs.

Another issue to consider is potential challenges with the synthetic chromosomes introducing biochemical incompatibilities or ‘chokepoints’. There are various biochemical pathways or reactions that are incompatible with each other inside a single cell. Microbial cells and communities deal with such biochemical incompatibilities by compartmentalisation either with separate cellular membrane-bound organelles or between distinct cells. Metabolic modelling of the synthetic microbiome might be useful for the design phase to minimise any potential biochemical incompatibility issues. Such incompatibilities may also contribute to the genetic instability of a synthetic metagenome chromosome. A promising solution would be to dynamically regulate incompatible pathways or genes so that they are not expressed at the same time. Depending on the application of the synthetic metagenome, this could be achieved using a variety of chemical, electric, or optogenetic induction systems^3,48^.

## Innovative strategies for overcoming problems

The most exciting feature of the Yeast 2.0 genome is the Synthetic Chromosome Recombination and Modification by LoxP-mediated Evolution (SCRaMbLE) system^49^. SCRaMbLE involves the inducible expression of a Cre recombinase that targets LoxP sites for recombination and results in gene loss, inversion, translocation and duplication. *LoxP* sites are included 3 bp 3′ of each non-essential gene’s stop codon, meaning that the final yeast genome will have thousands of *LoxP* sites, and therefore effectively infinite potential recombination combinations^8^. This means that although one synthetic yeast genome will be built, we can generate many millions of variants using SCRaMbLE. The number of unique genomes that SCRaMbLE can generate will be limited by lethality and the number of cells that can be cultured in a laboratory.

The main challenge in utilising SCRaMbLE is not in generating genetic diversity, but in applying relevant selection pressures or high-throughput screening of genomic variants. A further challenge then lies in understanding the genotype-phenotype relationship in SCRaMbLE isolates. Progress has already been made in these areas with demonstrations of SCRaMbLE for a range of improved biotechnological outcomes^50^. These examples were compatible with high-throughput screening as they relied on either metabolic pathways with colour outputs or on naturally growth coupled selection pressures such as heat and toxicity.

A synthetic metagenome will contain many genes that are non-functional, neutral, or deleterious for any given phenotype. An intriguing possibility would therefore be to ‘sculpt’ a synthetic metagenome using SCRaMbLE coupled to relevant selection pressures^51^. This approach would simultaneously remove unnecessary genes and optimise for the desired function. In the context of winemaking, the production of flavour molecules would be a commonly desired trait from a synthetic metagenome, but some other metabolic functions, with great industrial relevance, should be also considered when trying to reproduce the complexity of a whole wine yeast community, such as the metabolism of organic acids and complex nitrogen sources. At this point, it is necessary to highlight the first use of synthetic biology to improve the flavour production capacity of a *S. cerevisiae* wine strain which, containing a synthetic DNA cassette including genes from other yeast and plant species, it was able to produce, de novo, sensorially relevant quantities of the raspberry ketone aroma in the wine^52^.

Given that most flavour metabolites are not colourful, synthetic selection pressures would need to be applied to SCRaMbLE’d synthetic metagenome populations to isolate strains with desired flavour profiles. This can be achieved using biosensors that induce a survival response that is proportional to the concentration of target metabolite^53,54^. In contrast to colour-based selection, this approach has the throughput necessary to screen millions of unique SCRaMbLE’d genomes, which is necessary to sample even a small amount of the potential ‘biological solution space’ afforded by SCRaMbLE.

A parallel, complementary approach to the synthetic representation of a community microbiome in a single cell is to construct a consortium of synthetic microbes, where each individual contains a subset of the metagenome. While its likely impossible that a million genes from a complex microbial community microbiome could be expressed in a single synthetic yeast, it becomes a more tractable problem if that set of genes is expressed in, say, twenty synthetic yeasts with engineered dependencies as a simplified synthetic microbial community. Even for simpler microbial community microbiomes, such an approach might provide a solution for some of the potential issues discussed in this paper, if they are proving intractable. Already, there have been successful examples of engineered stable co-cultures between *S. cerevisiae* and *E. coli*^55^. To build such a synthetic microbial community would likely require engineering metabolic dependencies between the strains, along with corresponding transport (efflux and import), and signalling/sensing capabilities. Such a simplified synthetic microbiome echoes some of the advantages possessed by microbiomes in the real world where cooperative interactions among specialised community members overcome the physiological and metabolic constraints of individual organisms and allows mixed microbial populations to execute otherwise incompatible functions simultaneously.

## Wine ferments: a unique ecosystem to be condensed in a synthetic metagenome

Wine fermentation represents an ephemeral microbial ecosystem, defined by a deterministic microbial succession where a keystone species, *S. cerevisiae*, will end up as the dominant species. In fact, the relative abundance of *S. cerevisiae* in the community is a determinant for the taxonomic and functional diversity of the ecosystem (Fig. 2A). Thus, as the dominant species able to complete the wine fermentation process alone, it is assumed that the vast majority of keystone functions can be found in its genome. However, in all wine fermentations, especially in the early stages, *S. cerevisiae* co-exists and interacts with a great diversity of non-*Saccharomyces* yeast species (Fig. 2B, C), all of which contribute to the final complexity of metabolites that determine the flavour and quality of the end-product^5^. This poses a great opportunity to explore the biological challenge of capturing the key functions of the desirable members of a particular grape must’s yeast metacommunity in a synthetic metagenome of a *S. cerevisiae* wine strain.

It is known that some metabolic functions, representing important traits in wine ecosystem functioning are not effectively performed by *S. cerevisiae* under fermentative conditions (i.e. the uptake and use of certain nitrogen sources, the metabolism of some organic acids or the production of hydrolytic enzymes related to wine colour and aroma), and, within the non-*Saccharomyces* yeast community, a great inter-specific phenotypic variation has been found^56^. Identifying those keystone transcripts—without significant orthology in *S. cerevisiae*—and their host species will allow us to study their native transcriptional regulation for a latter introduction in *S. cerevisiae*’s genome (Fig. 1).

In this context, as part of the core community of wine fermentations, we can expect to find some already known flavour-active non-*Saccharomyces* species^57^ from genera such as *Candida*, *Debaryomyces*, *Lachancea, Kazachstania*, *Kluyveromyces*, *Metschnikowia, Pichia*, *Torulaspora* and *Wickerhamomyces* but also some other underexplored Basidiomycetous yeast species (e.g. *Solicoccozyma, Filobasidium* and *Papiliotrema*) (Fig. 2). Since the concept of ‘microbial *terroir’* was coined, it is accepted that the regional microbiota of grape musts determines the final metabolome of the resulting wines in a particular region^58^. However, there are conserved patterns of microbial species succession during the conversion of grape must into a wine, defined by largely aerobic, partially fermentative, and highly fermentative yeast species^57,58^. Thus, we hypothesize that a strong core metagenome can be modelled at a global scale, but also, regional metagenomes can be customized by including specific transcripts or sequence variants correlating with the flavour properties of wines from a certain region. Thus, it is a challenging yet exciting idea to explore the use of a synthetic *Saccharomyces* chassis for the construction of a designer yeast that will contain and regulate the expression of genes covering all the ecological functions found in multi-species spontaneous wine fermentations.

Finally, despite the fact that our proposal focuses on the wine ecosystem, some other yeast-based industrial processes (other food fermentations, biofuels bioreactors, etc), but also other bacteria-based industrial ecosystems (Waste Water Treatment Plants, Bioremediation-focused consortia) would be conceived within this perspective, that aims to combine the industrial potential of the revolutionary fields of synthetic genomics and systems ecology.
